# First-time voters in Türkiye: a thematic analysis from a political psychology perspective

**DOI:** 10.3389/fpsyg.2026.1976074

**Published:** 2026-10-28

**Authors:** Fatih Yaman, Musa Söztutar, Zübeyde Hande Yıldız Çeri

**Affiliations:** Department of Political Science and Public Administration, Manisa Celal Bayar University, Manisa, Türkiye

**Keywords:** first-time voters, political efficacy, political identity, political psychology, thematic analysis

## Abstract

Studies on young people’s participation in elections have largely focused on structural determinants, the formation of voting habits, or calculations based on rational choice. However, these approaches have limited scope when it comes to explaining how individuals experience and make sense of the moment they first become involved in electoral politics. To address this gap, this qualitative study is based on in-depth interviews with 26 young voters in Türkiye reflecting on their first voting experiences. The interviews were analyzed using reflexive thematic analysis with the support of MAXQDA, and the findings were interpreted through four analytical lenses: affective intelligence, social identity, cognitive dissonance, and political efficacy. The findings show that voting behavior is shaped not only by strategic considerations but also by how participants understand their political identities and perceive their ability to exert political influence. The study shows that casting a first vote is experienced as a psychologically meaningful moment of political self-understanding, extending accounts of youth political participation beyond structural conditions and strategic reasoning.

## 1 Introduction

This study examines how first-time voting is experienced and interpreted within the framework of political psychology. At a time when political disengagement, intergenerational divisions, and increasingly complex patterns of participation are becoming more pronounced, young people’s first encounters with electoral politics deserve closer attention. These experiences can be considered not only in terms of observable behavioral patterns but also through identity, cognition, and emotion. First-time voting is particularly important in this respect, as it represents an initial stage of direct participation through which individuals begin to position themselves in relation to the political system.

The existing literature on voting behavior has largely developed around three main research axes. Studies within the first axis highlight structural and developmental variables -such as resources, institutional arrangements, and life-cycle effects- in shaping electoral participation and voter preferences (Bhatti and Hansen, 2012; Plutzer, 2002; Smets and Van Ham, 2013). The second research axis conceptualizes voting behavior through rational choice models and limited information processing, focusing on decision-making mechanisms based on cost–benefit calculations and heuristic shortcuts (Blais, 2006; Lau and Redlawsk, 2001; Riker and Ordeshook, 1968). The third strand, grounded in political psychology, examines how emotions, identity formation, and perceptions of political efficacy shape political behavior (Henn and Foard, 2014; Huddy, 2001; Marcus et al., 2011; Valentino et al., 2011).

However, these approaches fall short in explaining how individuals experience their first time voting and what kinds of meanings they ascribe to that experience. While structural and rationalist studies rely largely on survey data, aggregate indicators, and observable behavioral outcomes, most research in the field of political psychology employs experimental or large-sample quantitative designs. Although these approaches make important contributions to explaining the determinants of voting behavior, they reveal less about how individuals construct and interpret their first encounters with electoral participation through their own narratives. Moreover, recent studies in Türkiye have examined young voters in relation to digital political participation and the use of media for political information (Oral, 2023; Sarıhan and Taşcıoğlu, 2024). These studies provide important evidence on young voters’ participation and media practices, but they do not focus directly on how young people interpret and make sense of the experience of voting for the first time. In this regard, this study seeks to overcome the mentioned weaknesses in theory and methodology by examining participants’ accounts through perspectives from political psychology and a thematic analytical approach.

Based on participants’ experiences of voting for the first time in Türkiye, this study examines how this experience can be viewed as a significant step in the formation of individuals’ political identities and their future political behavior. By analyzing the memory and perception of the participants of this experience, one can understand the way how the participation in the election process is perceived by the individuals. Special emphasis will be put on the emotions like fear, uncertainty, hope, and determination. The study is not aimed at measuring turnout or partisan behavior of participants. It studies how emotions, political identity, cognitive assessments, and political efficacy are revealed in the accounts of participants’ first voting experience. Voting is therefore approached not merely as a behavioral choice between candidates or parties, but as an experience through which individuals interpret their relationship with politics and their own capacity for political influence. Participants’ narratives form the basis of the analysis and are interpreted through theoretical perspectives from political psychology. Within this target group, the study draws on interviews with 26 university students from six universities in Türkiye. Participants were selected through maximum variation sampling to reflect variation in academic field, gender, and electoral experience.

## 2 Literature review

In the literature on voter participation, structural and developmental models -which have long sought to explain the phenomenon through resources, institutional arrangements and life-cycle-related effects- have gained prominence. Smets and van Ham (2013), highlighting the importance of political efficacy for electoral participation, state that “internal efficacy usually refers to the degree to which people think they can influence the government or policy outcomes, while external efficacy indicates the extent to which citizens perceive a government to be responsive to their interests. Both forms of efficacy are expected to increase turnout” (p. 355). Within this framework, however, psychological variables are largely treated not as dimensions of individuals’ lived experiences but as predictors of electoral participation. Developmental explanations similarly emphasize that voting behavior stabilizes over time. As Plutzer (2002) states, “voting behavior is, in part, a gradually acquired habit” (p. 42). Bhatti and Hansen (2012) further show that “when young adults leave home, the influence of their parents’ strong voting habits decreases while the weaker voting patterns among their peers have a greater impact” (p. 380). These studies therefore conceptualize first-time voting primarily in terms of structural conditions, political socialization and habit formation.

Recent research continues to show the importance of political socialization and electoral context during the early stages of participation. Fox (2024), using longitudinal data from newly eligible voters in the United Kingdom, found that childhood volunteering had little effect on turnout on average, but had a positive effect among young people whose parents were politically disengaged. Eckstein et al. (2024), using data collected before, during, and after national elections in Germany, the Czech Republic, and Sweden, found that political engagement increased during election periods. In the German sample, this increase was particularly pronounced among less engaged young people and first-time voters. These studies show that early electoral participation develops in relation to both previous political socialization and the context in which young people encounter elections.

Rational choice models, on the other hand, treat voting behavior as a decision-making process in which costs and benefits are weighed. Riker and Ordeshook (1968) argue that political participation should be theoretically explained rather than dismissed as irrational or inexplicable, stating that “the function of theory is to explain behavior and it is certainly no explanation to assign a sizeable part of politics to the mysterious and inexplicable world of the irrational” (p. 25). Subsequent work has emphasized bounded rationality in explaining voting behavior. Lau and Redlawsk (2001) show that voters rely on “heuristic-based information processing” rather than exhaustive evaluation (p. 968). At the macro level, Blais (2006) observes that “the main factors that affect variations in turnout are institutional variables” (p. 111). While these approaches shed light on strategic reasoning and decision-making under conditions of limited information, they remain limited in explaining the emotional ambivalence and identity-related uncertainty that may accompany voting for the first time.

Political psychology addresses this limitation by bringing emotion and identity more directly into the analysis of political behavior. Marcus et al. (2011) argue that when anxiety signals novelty, “reliance on habits diminishes and the brain shifts to deliberative consideration of the new information” (p. 333). Valentino et al. (2011) further demonstrate that “anger, more than anxiety or enthusiasm, will mobilize” participation (p. 156). Alongside emotion, identity also shapes the meanings individuals attach to political events, actors and preferences. As Huddy (2001) notes, “it is the meaning of American identity, not its existence, that determines its political consequences” (p. 130), showing that the political significance of identity depends on how that identity is understood.

In the context of young people’s political participation, Henn and Foard (2014) observe that young people often perceive “relatively few opportunities available for them to intervene effectively in formal political life” (p. 360).

Recent research in Türkiye has also examined the relationship between youth political participation and digital media. Öz Döm and Bingöl (2021), based on interviews with 30 young members of political parties and civil society, examined social media as a motivation for political participation. Toros and Toros (2022), using survey data collected after the 2018 general election, found that offline political participation was related to sharing political views online and exposure to alternative political ideas. Oral (2023) focused on how Generation Z uses media as a source of political information, while Sarıhan and Taşcıoğlu (2024) examined the digital political participation of 646 university students who were to vote for the first time in the 2023 general elections. More recent studies have also examined young voters’ perceptions of politicians’ social media use (Kocaman and Coşgun, 2024) and the relationship between social media use and political literacy among young voters (Balcı et al., 2026).

Within this framework, the study contributes to political psychology by addressing limitations in structural and rational models. It analyses participants’ narratives through a framework attentive to emotion, cognition and identity, thereby examining the psychological meanings associated with voting for the first time.

## 3 Theoretical perspectives from political psychology guiding the study

In addition to institutional and structural factors, individuals’ emotional orientations, cognitive evaluations and group affiliations also shape political behavior. Political psychology provides conceptual tools for examining these processes during moments of political entry such as voting for the first time. Rather than treating theory as a set of testable propositions or using these perspectives as predetermined coding categories, this study uses four perspectives -affective intelligence, social identity, cognitive dissonance and political efficacy- as analytical lenses for interpreting participants’ narratives. Their purpose here is not to repeat the literature review but to clarify how the inductively derived themes are interpreted at the level of meaning-making rather than prediction or measurement.

According to the affective intelligence perspective, emotions regulate attention when individuals face new or unfamiliar situations. Marcus et al. (2011) explain that “people have two distinct emotional systems: a dispositional system that incorporates people’s normal feelings about things and a surveillance system that manages their attention,” and that this surveillance system relies on emotions “most notably anxiety, fear, or uncertainty, to signal that something about the world is not routine and that conscious attention is necessary” (p. 324). Valentino et al. (2011) further distinguish between emotional states, noting that “Anger and enthusiasm, then, both lead us to take action” and that “we suspect anger and enthusiasm, more than fear or anxiety, are likely to push us to that choice” (p. 160). Brader (2005) similarly shows that “campaign ads can stimulate this pattern of behavior by cueing enthusiasm” (p. 398).

Social identity theory conceptualizes group membership as part of the individual’s self-concept. Tajfel (1974) defines social identity as “that part of an individual’s self-concept which derives from his knowledge of his membership of a social group (or groups) together with the emotional significance attached to that membership” (p. 69). Applied to partisanship, Greene (2004) argues that “partisan identification is an important social identity for many individuals” (p. 136). Mason (2015) further shows that sorting “has acted to increase the strength of political identities and has polarized mass political behavior” (p. 128).

Cognitive dissonance and motivated reasoning help explain how political commitments are maintained or adjusted after a choice has been made. Beasley and Joslyn (2001) emphasize that “voting represents decisional commitment in that it is clearly a behavioral act in support of a candidate” (p. 523), indicating that electoral choice carries psychological weight beyond the immediate act of voting. Taber and Lodge (2006) similarly demonstrate a “prior attitude effect such that attitudinally congruent arguments are evaluated as stronger than attitudinally incongruent arguments” (p. 755).

Political efficacy is examined here through participants’ perceptions of their capacity to understand politics and influence political outcomes rather than as a fixed personality trait. Karp and Banducci (2008) define electoral efficacy as “the feeling that one’s vote is potentially decisive” (p. 311), linking participation to the belief that individual political action can matter. Similarly, Niemi et al. (1991) define internal efficacy as “beliefs about one’s own competence to understand, and to participate effectively in politics” (p. 1407).

In light of these discussions, the study approaches first-time voting as an experience shaped by emotion, identity, consistency-seeking and perceptions of political competence. These perspectives guide the interpretation of how participants narrate their first voting experiences and position themselves in relation to the wider political system. Voting for the first time is therefore considered not only as a choice at the ballot box, but also as a psychological and social experience through which individuals begin to define themselves politically.

## 4 Methods

### 4.1 Research aim and questions

The forms of political participation and perspectives on political processes among first-time voters play a significant role in shaping future democratic functioning and policy-making processes. For this reason, taking into account the experiences, expectations and political orientations of younger generations is of critical importance not only for the development of democracy but also for strengthening social progress. In this context, examining the voting behavior of young people can contribute significantly to both policy-making and the academic literature.

In line with this aim, the study addresses the following research questions:

RQ1. How do participants describe and make sense of their first voting experiences?

RQ2. Which emotional, social, and cognitive processes play a role in the formation of their voting decisions?

RQ3. How do participants perceive the extent to which political actors and the political system respond to their expectations and demands?

RQ4. In what ways is the voting experience related to their developing political identities?

RQ5. What kinds of expectations, concerns, and emotional orientations do they express regarding the political future?

### 4.2 Research design

This study uses a qualitative research design and reflexive thematic analysis to examine how individuals perceive and interpret their first voting experiences. The constructivist worldview forms the philosophical basis of the study. In constructivism, reality is regarded as a socially constructed phenomenon. From this perspective, knowledge is co-created between the researcher and participants through interaction and interpretation. Accordingly, this study sought to gain rich and in-depth insights into participants’ subjective experiences by engaging them in open-ended conversations and analysing the resulting data inductively. Reflexive thematic analysis was considered suitable for this purpose because it enables the identification and interpretation of patterns of meaning across participants’ accounts while recognizing the active role of the researcher in the analytical process (Braun and Clarke, 2006, 2019).

The main purpose of the study is to understand participants’ accounts through their own perspectives, without using any pre-determined theoretical categories in the coding process. Accordingly, the analysis process was conducted using an inductive, theme-based approach that took into account the contextual complexity specific to each participant’s experience. The theoretical perspectives outlined earlier were used at the interpretation stage rather than as *a priori* coding categories.

### 4.3 Participant selection and data collection

In this study, participants were selected using maximum variation sampling, one of the purposive sampling strategies. This sampling approach was chosen with the aim of highlighting, within as broad a framework as possible, the diverse experiences, perspectives and ways of making sense of the first voting experience among young voters. Care was taken to ensure that participants were diverse in terms of academic field, gender and electoral experience, with the aim of reflecting the variations within Generation Z in the research. The sample consists of individuals born between 1997 and 2003 who had experienced voting for the first time and were able to reflect on that experience. This age range allows participants to be classified within the demographic boundaries of Generation Z. Ensuring diversity among participants is important for examining the different motivations, tendencies and patterns in the voting behavior of young people belonging to this generation in a more comprehensive manner (Table 1).

**Table 1:** Demographic information of participants.

Participants	University	Department	Year of birth	Gender	Elections voted
P1	Manisa Celal Bayar University	Political Science and International Relations	1999	Male	2018–2019–2023
P2	Manisa Celal Bayar University	Political Science and International Relations	1999	Female	2017–2018–2019–2023
P3	Manisa Celal Bayar University	Political Science and International Relations	1997	Female	2017–2018–2019–2023
P4	Manisa Celal Bayar University	Political Science and International Relations	2001	Male	2023
P5	Manisa Celal Bayar University	Political Science and International Relations	2003	Female	2023
P6	Manisa Celal Bayar University	Political Science and International Relations	2001	Female	2019–2023
P7	Hitit University	Physical Education and Sports Teaching	1999	Male	2019–2023
P8	Manisa Celal Bayar University	Political Science and International Relations	1997	Female	2019–2023
P9	Trakya University	Political Science and Public Administration	2001	Female	2023
P10	Trakya University	Political Science and Public Administration	2001	Male	2023
P11	Pamukkale University	Political Science and Public Administration	1998	Female	2019–2023
P12	Trakya University	Political Science and Public Administration	1999	Male	2018–2019–2023
P13	Pamukkale University	Political Science and Public Administration	2000	Female	2019–2023
P14	Bandirma Onyedi Eylul University	Political Science and Public Administration	2000	Male	2019–2023
P15	Pamukkale University	Political Science and Public Administration	2001	Male	2023
P16	Kastamonu University	Finance and Banking	2001	Female	2019–2023
P17	Hitit University	Coaching Training	2001	Male	2018–2019–2023
P18	Hitit University	Coaching Training	1997	Male	2017–2018–2019
P19	Hitit University	Coaching Training	2001	Female	2023
P20	Manisa Celal Bayar University	Political Science and Public Administration	2002	Female	2023
P21	Manisa Celal Bayar University	Political Science and International Relations	1999	Female	2017–2018–2019–2023
P22	Manisa Celal Bayar University	Political Science and International Relations	2000	Female	2019–2023
P23	Manisa Celal Bayar University	Political Science and International Relations	1999	Female	2017–2018–2019–2023
P24	Manisa Celal Bayar University	Political Science and International Relations	2000	Female	2019–2023
P25	Manisa Celal Bayar University	Public Relations and Publicity	2001	Female	2023
P26	Manisa Celal Bayar University	Public Relations and Publicity	2000	Female	2018–2023

The sample for this study consists of 26 participants studying in various academic departments at six different universities in Türkiye. Seventeen of the participants are women and nine are men. The interviews were conducted face-to-face between August 2023 and January 2024; during the data collection process, a semi-structured interview form was used, which allowed for the collection of in-depth data while maintaining thematic consistency among the participants. The interview guide covered participants’ understandings of Generation Z, their first voting experience, factors influencing their voting decisions, observations during the election process, expectations from politics and politicians, sources of political information, and their views on youth political attitudes and political responsiveness. Because the interviews were semi-structured, follow-up questions were used when necessary to clarify responses, obtain further explanation or examples, and explore issues raised by participants in greater depth. The complete interview guide is provided in Supplementary File 2.

Participants from other universities were interviewed in August 2023 while they were attending the summer school program at Manisa Celal Bayar University. Interviews with participants from Manisa Celal Bayar University continued in the following months.

The interviews were conducted without the participation of any third parties. No participants refused or withdrew from the study, and no repeat interviews were conducted. The interview form was pilot-tested with two individuals who met the inclusion criteria, and limited adjustments to the wording were made to enhance clarity and the flow of the discussion. The interviews lasted 30–40 min. With participants’ consent, all interviews were audio-recorded. The interviews were conducted jointly by the first and third authors. The first and second authors are male academics in the field of political science, while the third author is a female postgraduate student in the same field. All three researchers have experience in qualitative research.

The fact that some participants had voted in more than one election added analytical depth to the research. This made it possible to examine not only participants’ accounts of their first voting experiences but also their reflections on whether and how their perspectives had changed in subsequent electoral contexts. Although the study is not based on a longitudinal research design, participants with multiple electoral experiences were able to draw comparisons between their first and later voting experiences. These participants were not treated as a separate analytical group; rather, their later experiences provided an additional point of reference for interpreting their first voting experience.

The researchers’ academic backgrounds in political science and their shared academic interest in youth political participation were considered a positioning factor that needed to be taken into account throughout the research process. Although this positionality was seen as an opportunity that could foster sensitivity in the interpretation of participants’ narratives, it was also recognized that it could entail a risk of interpretative bias; consequently, reflexive awareness was maintained throughout the data collection and analysis processes. All interviews were conducted in the first author’s office at the Faculty of Economics and Administrative Sciences, Manisa Celal Bayar University. The office provided a quiet and private setting in which the interviews could be conducted without interruptions. Participants were informed that there were no “right” or “wrong” answers and were encouraged to express their views freely. Some participants from Manisa Celal Bayar University were acquainted with the researchers in an academic context, whereas participants from other universities had no prior relationship with the research team.

### 4.4 Data analysis and theme development

The collected data were analyzed using reflexive thematic analysis. Once the verbatim transcripts had been imported into MAXQDA 26, the texts were reviewed several times to support familiarization with the data, and the coding process was gradually refined in line with these repeated readings. Initial codes were developed inductively and closely related codes were subsequently grouped into descriptive categories. These categories were then examined in relation to broader patterns of meaning during the development of candidate themes, which were reviewed and refined through repeated movement between coded segments, individual transcripts and the dataset as a whole. This process enabled the analysis to move beyond the identification of individual codes toward the interpretation of broader patterns of meaning relevant to participants’ first voting experiences. Table 2 provides a concrete example of the coding process followed in this study.

**Table 2:** Example of the coding and theme development process.

Themes	Categories	Codes	Quotes
Theme: Generational self-perception and identity	Category: Cognitive and affective representations of generation Z	*“Born into technology”*	*“Generation Z is a generation born into technology. This is the biggest difference between them and other generations.” (P4)*
Theme: Psychological dynamics of political engagement	Category: Perceived political agency and internalized civic norms	*“Democratic right of citizenship”*	*“First and foremost, the reason I voted was because it is my right as a citizen, and I felt that I had to use it.” (P8)* *“I exercised my vote as my democratic right.” (P12)*

In addition to the interview transcripts, field notes and analytical memos were kept on a regular basis to enable the tracking of analytical assessments that emerged during the data collection and coding process. These records helped the researchers to preserve the train of thought that emerged during the process and to pursue the interpretation process in greater depth.

The frequency figures and participant-based code matrices represent two different units of analysis. The frequency figures show the total number of coded segments; therefore, the same participant may contribute more than one coded segment to a particular code when the issue appears in different contexts within the interview. In contrast, the participant-based matrices indicate whether a code appears in each participant’s transcript; each participant is counted only once for a given code (shown as 1), regardless of the number of coded segments in that transcript. The resulting totals may therefore differ. These frequencies are presented descriptively and were not treated as indicators of the analytical importance of particular codes or themes.

### 4.5 Reflexivity and analytical rigor

Analytical rigor was supported through repeated engagement with the interview transcripts, analytical memoing, and ongoing reflection on the development of codes and themes. In line with the reflexive thematic approach, coding was not treated as a process aimed at establishing a single objectively correct interpretation of the data. Instead, the researchers’ interpretive role was acknowledged throughout the analysis.

The first and second authors engaged in iterative analytical discussions during the development and refinement of themes. These discussions were used to consider alternative interpretations, question emerging assumptions, and refine the boundaries and meanings of the themes rather than to establish inter-coder agreement. All stages of the analytical process were documented to support transparency and consistency between the research questions, the data and the final thematic structure.

Selected participants were contacted again during the analytical process to obtain their reflections on preliminary thematic interpretations. These responses were considered as an additional interpretive perspective rather than as confirmation of a single correct interpretation. Data collection concluded after 26 interviews, by which point later interviews were producing increasingly recurrent accounts. This was treated as a pragmatic indication of dataset adequacy rather than as evidence that all possible themes had been exhausted. Throughout the study, the Consolidated Criteria for Reporting Qualitative Research (COREQ) checklist was used as a basis to support the transparency of the reporting process, researcher reflexivity and analytical rigor (Tong et al., 2007; see Supplementary File 1).

### 4.6 Ethical considerations

This research was approved by the Manisa Celal Bayar University Social and Human Sciences Scientific Research and Publication Ethics Committee (Decision No: 2022/10; Ref. No: E.447045). All procedures were conducted in accordance with ethical guidelines for social science research. Given the political sensitivity of the topic, particular attention was paid to ensuring a voluntary and secure research environment. Prior to each interview, participants were informed about the purpose of the study, their right to withdraw at any time, and the confidentiality of their responses. Written and verbal informed consent were obtained.

Participants were assigned numerical identifiers (e.g., P1, P2), and identifying information was removed during transcription. Audio recordings and transcripts were stored in password-protected files accessible only to the research team. To reduce potential social desirability pressures, participants were reminded that participation was voluntary, that their responses would remain confidential, and that there were no right or wrong answers. Interviews were conducted in a quiet and private office setting.

### 4.7 Limitations

As the data are based on self-reports relating to politically sensitive experiences, there is a possibility that social desirability may have influenced participants’ narratives. Although confidentiality, voluntary participation, and the absence of “right” or “wrong” answers were emphasized throughout the interviews, this possibility cannot be entirely ruled out. In reflexive thematic analysis, researchers’ perspectives form part of the interpretive process; accordingly, the themes presented in this study reflect an interaction between participants’ accounts and the researchers’ analytical engagement with the data.

The sample consisted entirely of university students, which limits the transferability of the findings to young people outside higher education. In addition, some participants from Manisa Celal Bayar University were acquainted with members of the research team in an academic context, and all interviews were conducted in the first author’s university office. This institutional proximity may have influenced how some participants expressed politically sensitive views, even though confidentiality and voluntary participation were emphasized throughout the interviews.

Because some participants were recalling first voting experiences that had occurred several years earlier, their accounts may also have been shaped by retrospective recall and subsequent electoral experiences. In future research, expanding the sample to include individuals outside the university context and examining the dynamics identified here through comparative, longitudinal or mixed-methods designs could enable a more detailed assessment of how the findings are shaped in different social and electoral contexts.

## 5 Findings

In this section, the analysis of data obtained from the interviews revealed that the participants’ experiences were organized around four main themes. The themes address the research questions in overlapping ways rather than corresponding to them on a one-to-one basis. Generational Self-Perception and Identity relates primarily to participants’ developing political identities and their orientations toward the future (RQ4 and RQ5). Psychological Dynamics of Political Engagement addresses how participants made sense of voting and the emotional, social, and cognitive processes involved in their decisions (RQ1 and RQ2). Political Information and Aspirational Orientations concerns both political information-seeking and participants’ expectations of political actors and the political system (RQ2, RQ3, and RQ5). Youth Expectations and Perceived Political Responsiveness relates most directly to participants’ perceptions of responsiveness and their expectations regarding the political future (RQ3 and RQ5). The first of these, Generational Self-Perception and Identity, focuses on the common characteristics through which Generation Z participants define themselves and how they ascribe meaning to their generational identity. Within this framework, the category Cognitive and Affective Representations of Generation Z reflects both the participants’ cognitive assessments and emotional orientations regarding their own generation. Participants’ narratives suggest that generational self-perception also provides a context for how they position themselves politically. When assessed from the perspective of social identity theory, belonging to Generation Z goes beyond a simple demographic definition for the participants. This sense of identity is related to how young people analyse the political world, access sources of political information and position themselves within a political context that also includes their expectations of the political system. Within this framework, the close relationship that this generation maintains with technology, their ability to adapt rapidly to change and the anxieties they harbor regarding their future are not merely labels that define this group. These characteristics also accompany how participants perceive themselves as a generation and interpret their political environment.

In the second theme, “Psychological Dynamics of Political Engagement”, participants’ accounts of voting for the first time are examined through the emotional, cognitive and social processes associated with political participation. This theme comprises two mutually reinforcing categories: “Perceived Political Agency and Internalized Civic Norms” and “Social Influence, Value Orientations, and Political Evaluations”. The first concerns how participants understand voting as a civic right, a form of political agency and a possible means of expressing demands for change. The second concerns how the social environment, personal and collective values, candidate-related evaluations and broader political considerations are reflected in participants’ voting preferences. When these two elements are considered together, the experience of voting for the first time cannot be assessed solely as an individual and rational cost–benefit analysis. Emotions such as hope, disappointment and anxiety—as embodied in the statements of the participants—also accompany political participation. In this context, voting decisions are described through a combination of deliberation, social influence, identity-related orientations and emotion.

The third theme of the study, “Political Information and Aspirational Orientations”, examines both how participants engage with sources of political information and what they expect from political actors and the political system. This theme is further divided into two categories. The first, “Media-mediated Political Learning and Information-Seeking”, focuses on the sources through which participants follow political developments and seek political information. The second, “Expectations, Needs, and Perceptions of Political Trust”, examines participants’ expectations concerning political representatives and broader social and political conditions, together with how these expectations relate to their perceptions of political trust and responsiveness. Considered together, these categories show that participants’ orientations toward politics are shaped both by the information environments through which they encounter politics and by their expectations concerning recognition, justice, freedom and the future.

The fourth theme, “Youth Expectations and Perceived Political Responsiveness”, focuses on young people’s expectations concerning a more responsive and inclusive political environment. The category “Demands for Responsive Representation” encompasses participants’ proposals regarding how political parties, candidates and representative mechanisms could establish more effective relationships with young people. This theme demonstrates how participants articulate expectations of being understood, represented and responded to by political actors and institutions. Their proposals concern not only practical political demands but also the conditions under which they believe political participation and change could become more meaningful.

Figure 1 presents the category *Cognitive and Affective Representations of Generation Z* under the theme *Generational Self-Perception and Identity*. The related views were organized into nine codes. *Born into technology* appeared in 15 coded segments, while *Inquisitive, research-oriented, conscious* (7) and *Open-minded/innovative thinking* (6) were also common in participants’ descriptions. *Hopeful* was coded in six segments, while *Living with uncertainty and anxiety* appeared in five. These accounts show that participants described their generation not only through technology and openness to change, but also through different emotional orientations toward the future. Other codes included *Open to influence, changeable* (4), *Experiencing an economic crisis* (3), *More sensitive* (2), and *Freedom-seeking* (2).

**Figure 1:**
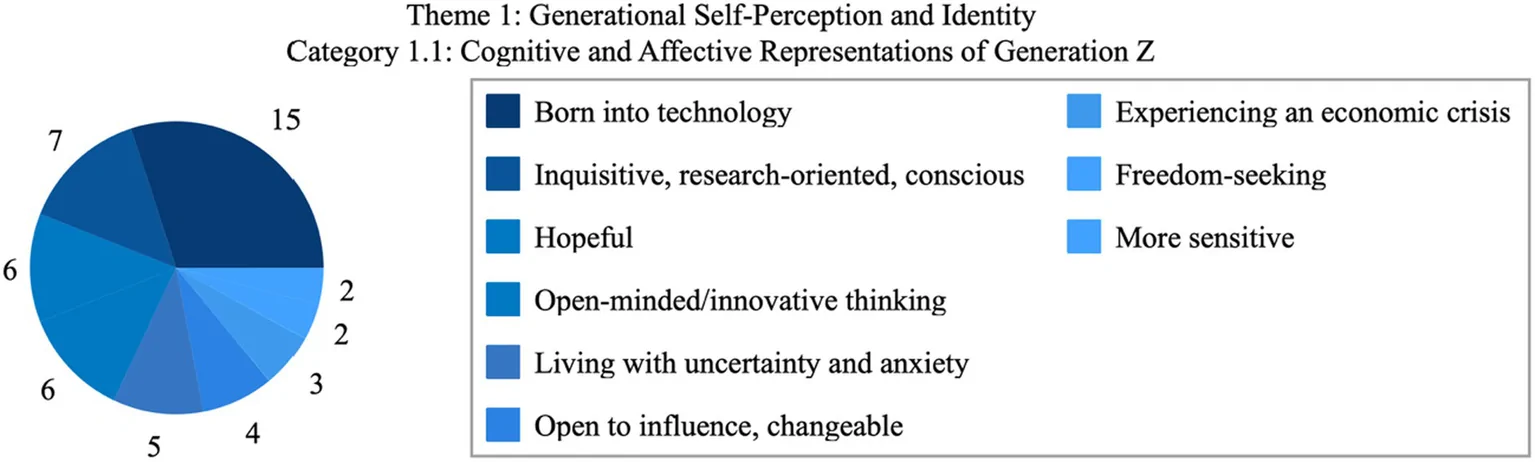
Distribution of coded segments in the cognitive and affective representations of generation Z category.

The participant-based coding matrix shown in Figure 2 indicates that these characteristics appeared across different interviews rather than being limited to a small number of participants. Technology was a common reference point, but participants did not attach the same meaning to their generational position. Some associated it with greater awareness and political engagement, while others emphasized uncertainty or expressed optimism about the capacity of their generation to bring about change.

**Figure 2:**
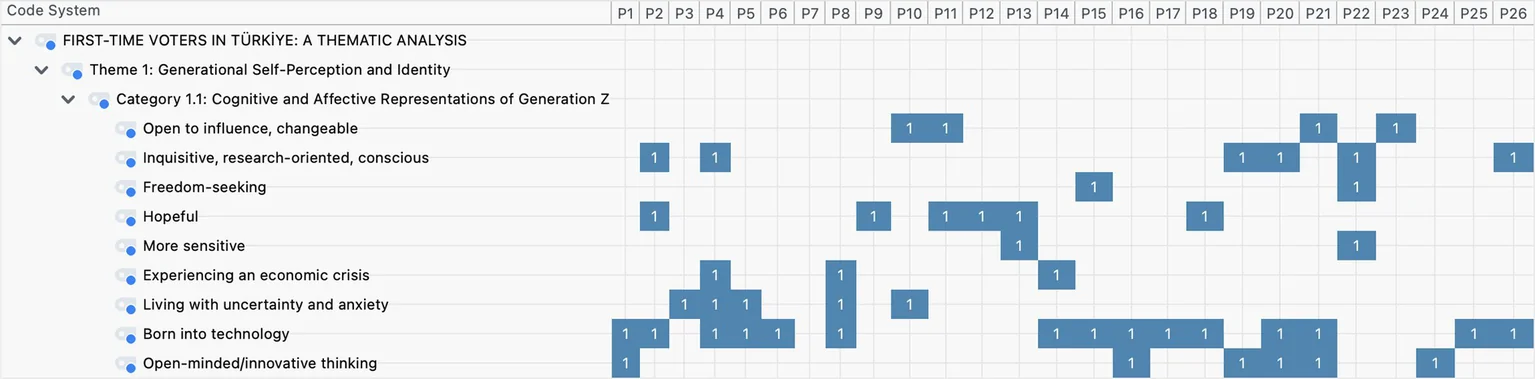
Participant-based code matrix for the category cognitive and affective representations of generation Z.

One participant linked technological familiarity with greater awareness of politics:

*“Contrary to what is generally assumed, I think we are a more conscious generation, born into technology. Therefore, we follow current events, the past, and the future more closely. I think Generation Z is more conscious and more connected to civic duties and politics.” (P2)*

Another participant emphasized uncertainty:

*“Based on my close circle, we are an indecisive and excited generation that does not quite know what it wants or what it wants to do. I agree with the view that we came into the worst period. We are unlucky and indecisive.” (P3)*

A more optimistic account associated the generation with the possibility of change:

*“Generation Z represents a revolution for me compared with other generations. There are many differences in technology, communication and transportation that the previous generation did not have. This makes me think that this generation can change Türkiye. Generation Z is the generation with that opportunity.” (P18)*

Figure 3 presents the category *Perceived Political Agency and Internalized Civic Norms* under the theme *Psychological Dynamics of Political Engagement*. Participants’ views were organized into five codes. *Democratic right of citizenship* was coded in 12 segments and *Expectation of change/innovation* in eight. *Personal values and identity* (4), *Legitimate expression of dissent* (3), and *Freedom of thought* (2) added different meanings to why participants considered voting important. Voting was therefore described not only as a civic right, but also as a means of expressing a political position, exercising individual agency, and seeking change.

**Figure 3:**
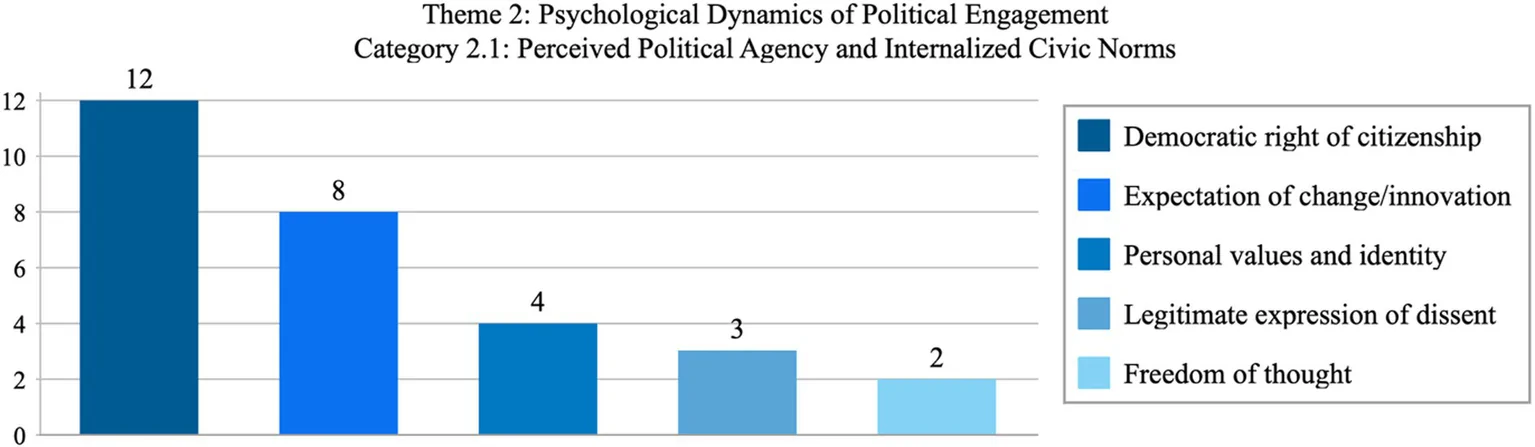
Distribution of coded segments in the perceived political agency and internalized civic norms category.

The participant-based matrix shown in Figure 4 reflects differences in how political agency was experienced. For some participants, voting combined civic responsibility with the possibility of expressing dissatisfaction. Others associated voting more directly with becoming an individual or with expectations that political change could occur.

**Figure 4:**
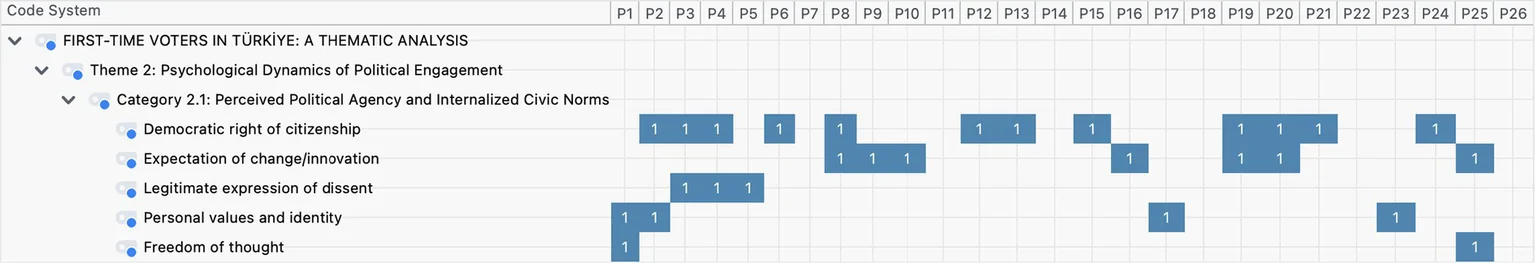
Participant-based code matrix for the category perceived political agency and internalized civic norms.

One participant described both the legitimacy and the limits of individual voting:

*“I voted out of civic awareness. Voting was the most legitimate way for me to express my reaction to issues I was uncomfortable with. It was my first time and I was excited. After voting, I realized that one vote has little effect. If there is going to be change in society, it has to be collective, not individual.” (P4)*

Another gave voting a more personal meaning:

“I voted to feel that I was an individual.” (P17)

A different account connected voting directly with hope for political change:

*“I still had some faith in democracy and I voted to change things. I thought change would happen. I was hopeful.” (P25)*

*“It was my first time voting. Voting for me was like looking for an option within a lack of options. I thought there should be a complete change. Seeing the same names repeatedly kept leading to the same things. I think this was helplessness.” (P10)*

Figure 5 presents the category *Social Influence, Value Orientations, and Political Evaluations* under the theme *Psychological Dynamics of Political Engagement*. *Influence of family* (16) and *Influence of social media* (14) were frequently represented in the coded material. *Social environment* and *Promises made by candidates* were each coded in ten segments. Candidate characteristics, ideological orientations, nationalism and religion, economic conditions, and participants’ broader worldviews also appeared in their accounts. The interviews show that political preferences were related both to social influences and to participants’ own evaluations of candidates, values and political conditions.

**Figure 5:**
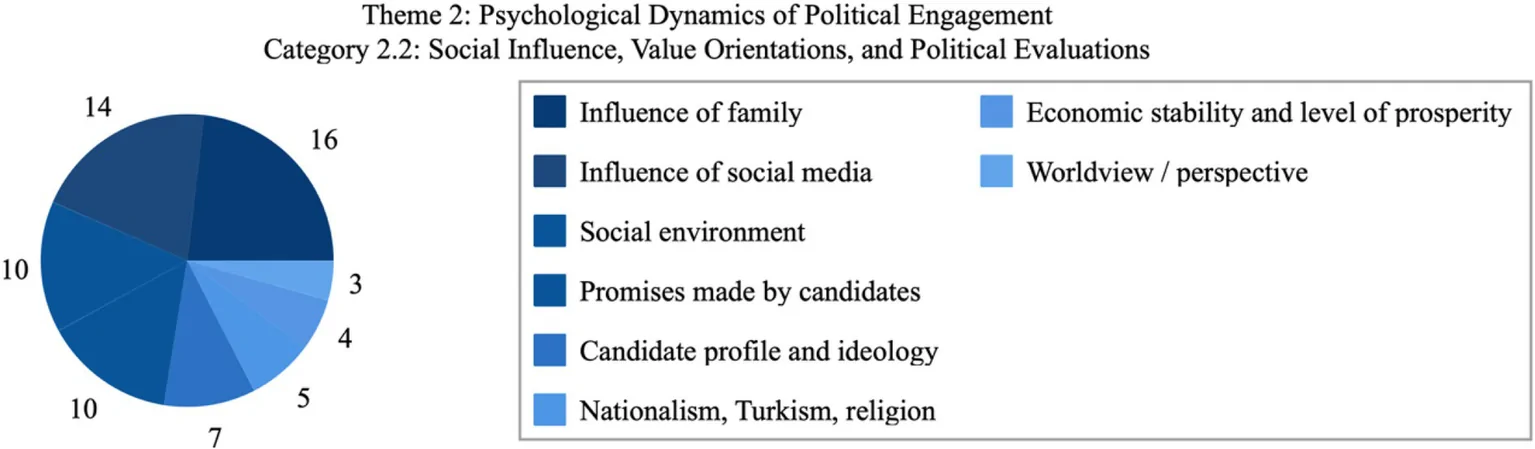
Distribution of coded segments in the social influence, value orientations, and political evaluations category.

The participant-based matrix shown in Figure 6 indicates that these influences were distributed differently across participants. Family and social environment were important in some accounts, but participants also described later processes of questioning, candidate evaluation and exposure to political information. Value orientations were more decisive for others.

**Figure 6:**
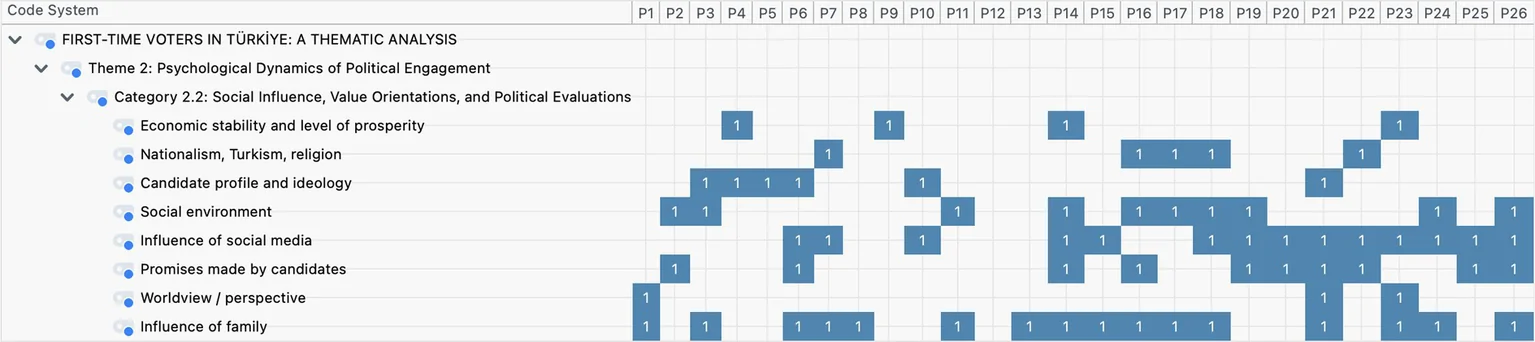
Participant-based code matrix for the category social influence, value orientations, and political evaluations.

One participant described family as an early influence without treating it as fully determining later political views:

*“Candidates’ promises and ideology have an effect, of course, but they do not completely change things. Family lays a certain foundation from childhood. But my views really took shape in my youth; if I had to put a number on it, family twenty percent, myself eighty percent.” (P14)*

Another emphasized the evaluation of campaign promises and information encountered through media:

*“I already had a political view, but I examined the candidates’ promises. The media is also important because it puts the positive and negative sides of each candidate in front of us. It makes it easier for us to see and question them. I tried to find more objective comments through the media.” (P20)*

*“Social media influences me and my friends in lasting and different ways. But when we look at our families, they are strongly attached to another party. We are first shaped by our families’ views, and then we can change through the influence of social media and politicians.” (P6)*

A different account placed greater weight on established ideological and value orientations:

*“I never saw the economy as a determining factor in my voting behavior. We are a Muslim country; religion, Turkism and nationalism affect me. I vote for whoever holds this view.” (P7)*

Figure 7 presents the category *Media-mediated Political Learning and Information-Seeking* under the theme *Political Information and Aspirational Orientations*. *Instagram-Twitter* was coded in 22 segments, *Various YouTube channels* in 18, and *Following television* in eight. Digital platforms therefore occupied an important place in participants’ accounts of political information. The interviews also show that these platforms were used in different ways. Some participants followed an initial piece of content with further reading, while others moved between television, social media and YouTube or deliberately sought views different from their own.

**Figure 7:**
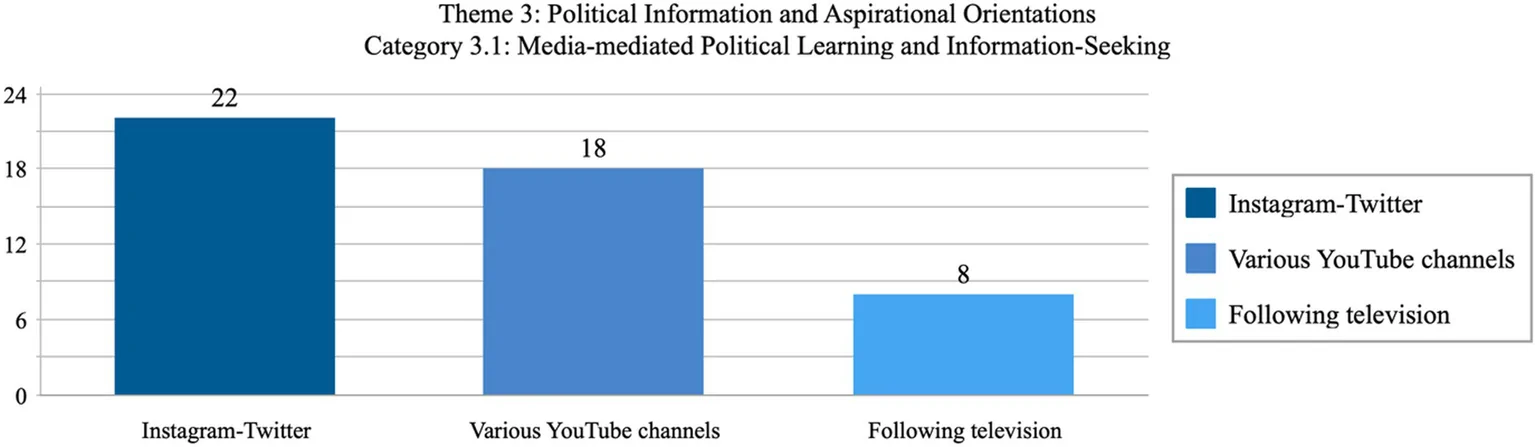
Distribution of coded segments in the media-mediated political learning and information-seeking category.

The participant-based matrix shown in Figure 8 indicates that social media and YouTube appeared across a broad part of the sample, while television remained part of some participants’ information environments. The accounts suggest that media use involved more than choosing a single platform; participants often described moving between sources and evaluating the information they encountered. The platform codes were based on sources mentioned by participants during the interviews rather than on a predefined list of social media platforms.

**Figure 8:**
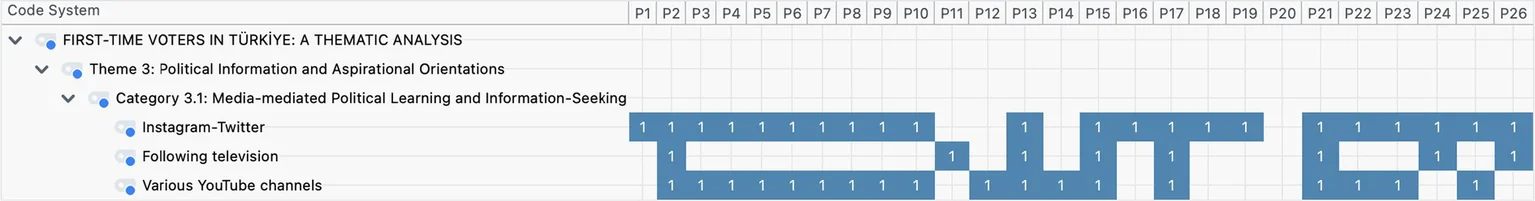
Participant-based code matrix for the category media-mediated political learning and information-seeking.

One participant explained how a short social media video could lead to further political information-seeking:

*“I see a two-minute video and think, ‘that was a good speech.’ Then I get curious about the person and go to their social media account. I try to read their posts, their views on events, and the texts they write. I also try to learn about the ideologies they believe in.” (P1)*

Another described moving between traditional and digital media:

*“I use mass media, mainly news channels. But we follow things that are not shown on the news through social media. I follow Twitter or news sites. I also watch YouTube.” (P21)*

A third account included an explicit attempt to encounter views beyond the participant’s own political position:

*“I mostly follow Twitter because that is where most news first appears. I also follow Instagram, and sometimes television and radio. I want to follow news channels even when they do not reflect my own views because I want to see the other side. Sometimes I feel as if I am in an echo chamber because I end up following more people who share my own views.” (P24)*

*“I use Instagram and Twitter. I think following their own accounts rather than fan accounts is healthier, because fan accounts tend to go back to older events and present past mistakes more negatively. I think they should all be followed through their own accounts.” (P16)*

Figure 9 presents the category *Expectations, Needs, and Perceptions of Political Trust* under the theme *Political Information and Aspirational Orientations*. *Economic freedom* was coded in 12 segments and *Personal development/quality education* in eight. Employment and individual freedom were each represented in seven segments, while justice and honest and ethical decision-making appeared in six. Other codes concerned security, meritocracy, transportation, accountability and participation, being understood, fulfillment of promises and political change. Participants’ expectations therefore included both material conditions and the way political institutions and representatives were expected to respond to citizens.

**Figure 9:**
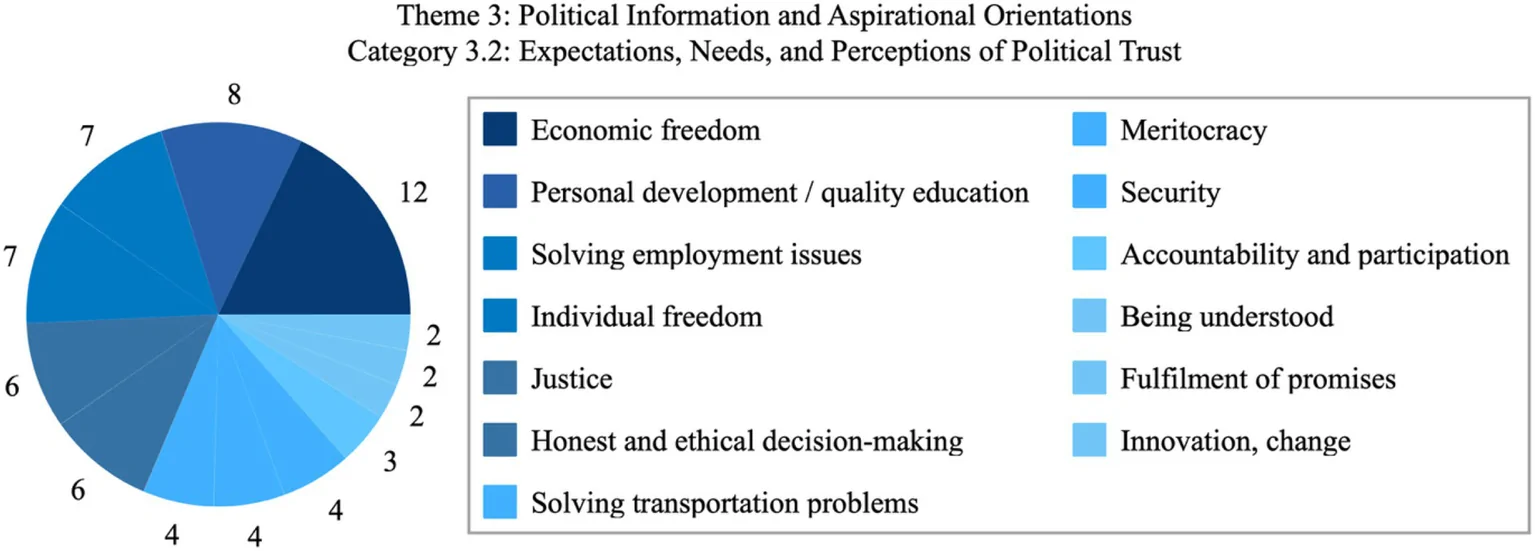
Distribution of coded segments in the expectations, needs, and perceptions of political trust category.

The participant-based matrix presented in Figure 10 shows that these expectations were not concentrated in a single area. Economic conditions and education appeared alongside justice, freedom, accountability, security and communication. This variation is important for understanding political trust here as a perception related to several aspects of participants’ relationship with the political system rather than as a single attitude.

**Figure 10:**
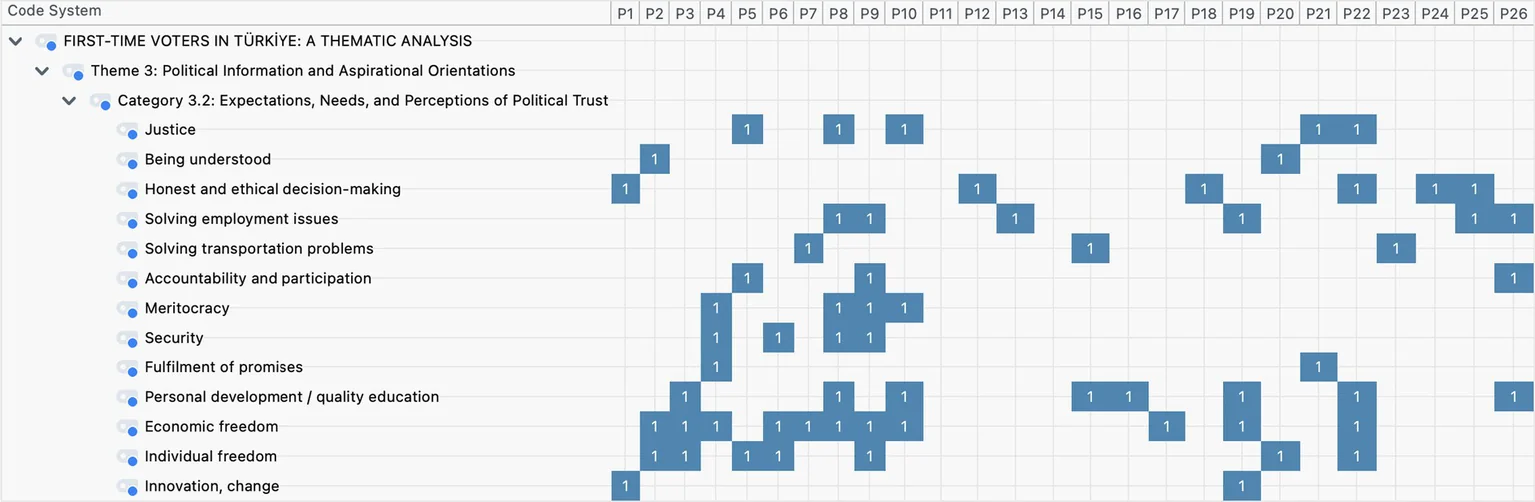
Participant-based code matrix for the category expectations, needs, and perceptions of political trust.

One participant focused specifically on transparency and accountability:

*“They need to be more open. We want to see where the taxes we pay go. When we look for an answer to this question, we face various accusations. There should be an accountable, transparent and open structure.” (P5)*

Another combined economic expectations with security, education and a desire for support from elected representatives:

*“Generation Z wants economic comfort and prosperity. They want security, comfortable spaces and dormitories, and more educational opportunities. They want to know that the person who is elected stands behind them, protects them and is there for them.” (P8)*

In another account, the focus was on justice, freedom, and political communication:

*“I think they expect a just and free environment. I want us to be given a little more freedom. I also want the language used toward us to be softer because there is a real lack of communication.” (P22)*

*“At least, we expect to be in a better position than we are now, especially in terms of the economy and education. We expect things to change. We have serious problems with education, housing, and finding a job after graduation.” (P19)*

Figure 11 presents the category *Demands for Responsive Representation* under the theme *Youth Expectations and Perceived Political Responsiveness*. *Politicians should value and understand young people* was coded in nine segments, *Concerns about the future should be alleviated* in seven, and *Politicians should improve themselves* in six. Participants also referred to opportunities to go abroad, equal living conditions, access to technology, education, basic needs, economic policies and opportunities for young people to participate in politics. These demands concerned both young people’s living conditions and the relationship they expected political actors to establish with them.

**Figure 11:**
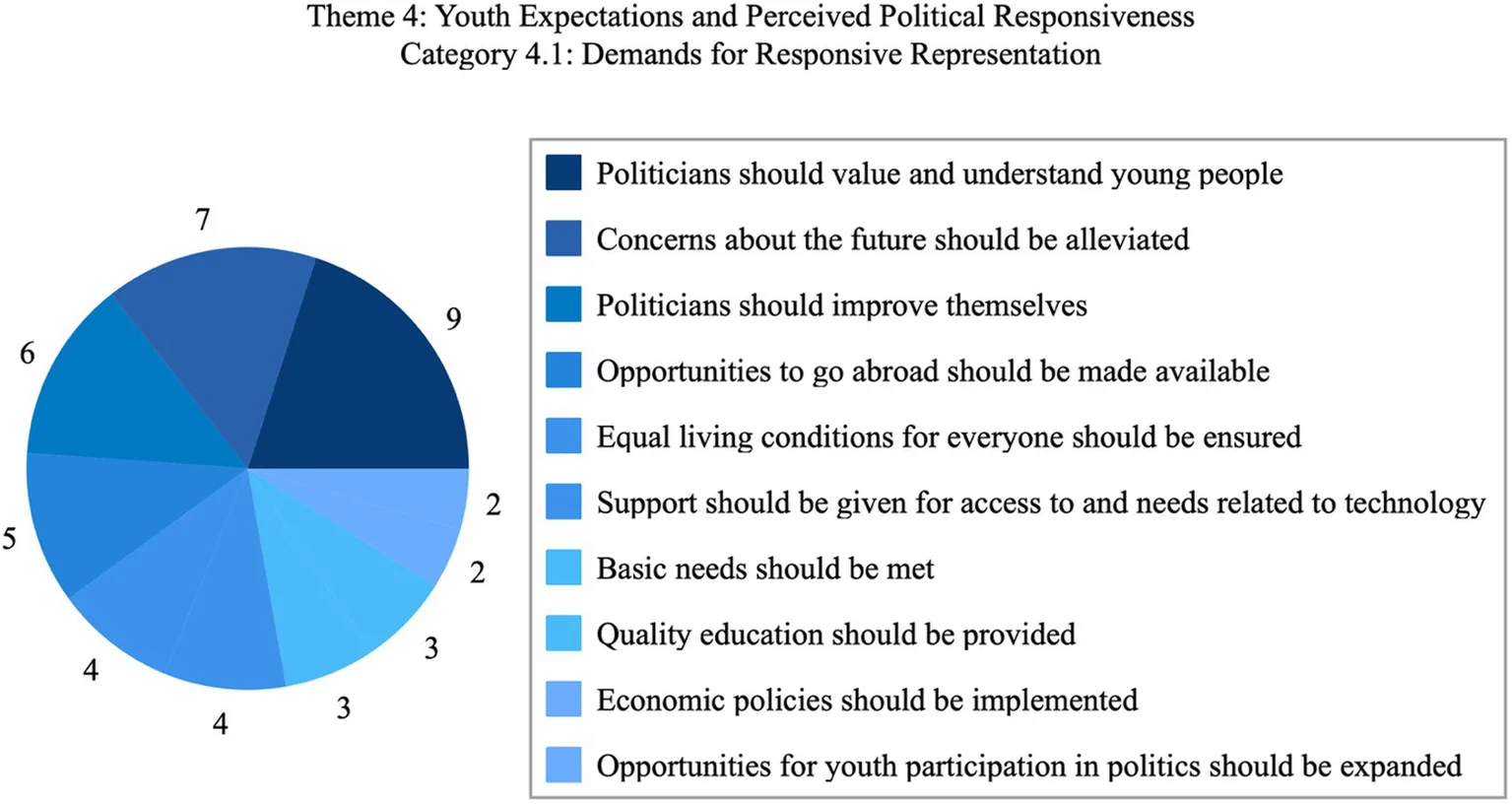
Distribution of coded segments in the demands for responsive representation category.

The participant-based matrix mentioned in Figure 12 reflects the different forms that these demands took. Some accounts centered on employment, economic conditions and future uncertainty. Others focused on political communication, being listened to and creating more opportunities for young people to participate.

**Figure 12:**
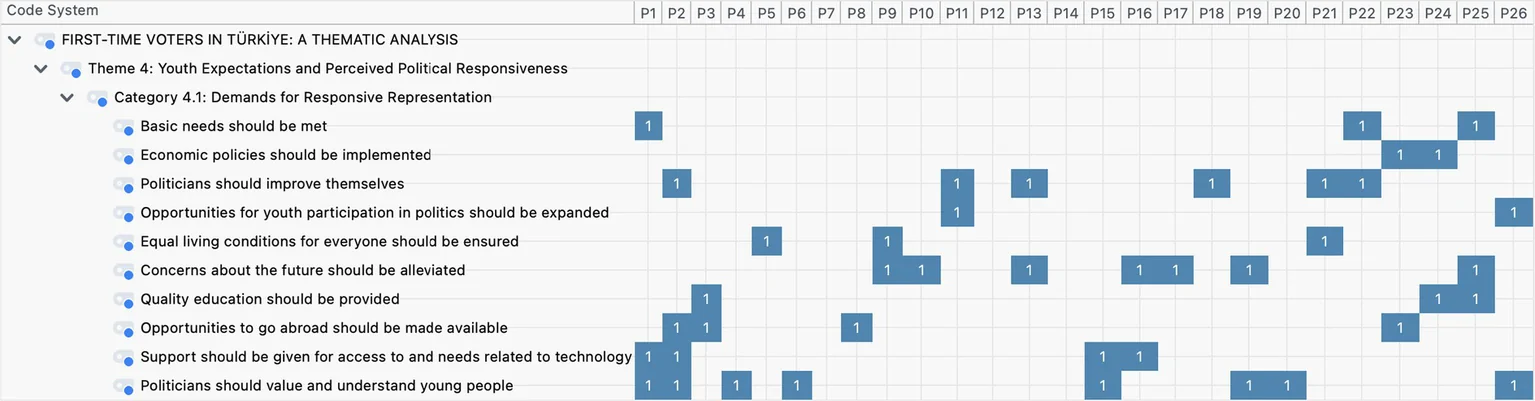
Participant-based code matrix for the category demands for responsive representation.

One participant connected future anxiety with unemployment and unequal opportunities:

*“I think the reason Generation Z becomes depressed is completely related to unemployment and the economic situation. I will graduate next year, but I do not know what I am going to do. There is a need to ensure that everyone can live under equal conditions.” (P9)*

Another focused on the style of political communication:

*“My complaint is that they sometimes speak by shouting and raising their voices. I think it could be calmer. I know that my friends think the same way.” (P13)*

A third account placed greater emphasis on listening and participation:

*“They should listen to young people. They should adopt a participatory approach. A platform could be created for young people where their suggestions could be collected. The most important thing is that young people are listened to.” (P26)*

*“Personally, politicians should improve themselves. For example, do they read books? I think they speak too much in colloquial language, even at a lower level. They should focus more on English.” (P11)*

## 6 Discussion

As the research findings also show, for young voters, voting for the first time does not simply mean participating in the electoral process. According to data from the interviews, this first experience represents a process in which individuals assess how they position themselves within the political system, what emotions accompany their voting behavior, and whether the choices they ultimately make align with their perception of their own political reality. When approached from this perspective, the notion that the first voting experience is based solely on the resources available to voters in this group or on the strategic calculations they make is incomplete (Blais, 2006; Riker and Ordeshook, 1968). When the first election day arrives, the young voter begins to reflect on their political identity and enters a process of making sense of their choices in this arena, thereby beginning to question the nature of the direct or indirect relationship they have established with the political system.

During the research process, participants described their generation through expressions such as “born into technology”, “questioning” and “living with uncertainty”. These shared definitions suggest that generational belonging is not merely demographic but is also connected to common historical and socio-economic conditions. In line with social identity theory (Greene, 2004; Mason, 2015; Tajfel, 1974), the findings indicate that generational identity may provide a shared point of reference for political self-positioning, even where strong party affiliation is absent.

According to the participants’ statements, emotions do not influence first-time voting in a single direction. Affective intelligence theory associates anxiety with greater attention to unfamiliar situations and links emotions such as anger and enthusiasm to political mobilization (Marcus et al., 2011; Valentino et al., 2011). In this study, however, anxiety, hope, optimism and disappointment often appeared together and were closely tied to economic insecurity, distrust of institutions and limited political options. This suggests that the political effects of emotion depend strongly on the context in which these emotions are experienced.

In addition, when young voters determine their political preferences, they do more than simply express their support for a single candidate or party. They also seek to establish a connection between their choice and their own political stance. Within this framework, political information may be processed in a biased manner, serving to preserve individuals’ previous decisions and allegiances. Rather than evaluating new political developments impartially, individuals may interpret them through the lens of their existing beliefs and commitments (Taber and Lodge, 2006); such commitments may also influence their subsequent political evaluations (Beasley and Joslyn, 2001). The participant’s description of voting as “searching for an option within a lack of options” suggests that first-time voting may involve an effort to reconcile political preferences with a restricted set of electoral alternatives. In fact, such statements do not fundamentally indicate inconsistency. They also reveal the effort of political identities, which are still developing around the first voting experience, to find a balance with the electoral alternatives available within the existing system. In this context, voting for the first time can be understood as a process of political self-positioning in which individuals’ self-definition and political preferences mutually shape one another.

Although political efficacy remains visible within the research findings, it also appears to rest on relatively fragile ground. The classical distinctions in the literature between internal and external political efficacy concern individuals’ beliefs regarding their own political competence and the responsiveness of the political system to citizens’ expectations (Karp and Banducci, 2008; Niemi et al., 1991). The fact that participants regard voting as a democratic right points primarily to an internalized civic norm rather than, by itself, to a perception of internal political efficacy. At the same time, participants’ assessments of whether their political actions can make a difference, together with references to systemic stagnation and limited responsiveness, reveal the conditional nature of political efficacy within their accounts. The act of voting is not only a reflection of efficacy but is itself involved in its negotiation. Among the participants, efficacy therefore appears as a dynamic perception rather than a fixed predictor of voting, shaped by context and by their electoral experiences.

The findings also have practical implications for political communication and youth engagement. Participants’ accounts suggest that engagement with young voters is related not only to election campaigns but also to access to political information, opportunities to express their concerns, and perceptions that political actors respond to their expectations. For political parties, public institutions, and youth-oriented civil society organizations, these findings point to the relevance of providing accessible political information and creating channels through which young people can express their priorities and receive responses. The findings also suggest that youth engagement should not be approached through a single communication platform, as participants described moving between social media, YouTube, television, and other sources when following political developments. This is consistent with recent research in Türkiye showing links between social media use, political participation, and political information among young people (Oral, 2023; Toros and Toros, 2022). Recent qualitative research also shows that young voters evaluate not only the content of political communication on social media but also how politicians communicate through these platforms (Kocaman and Coşgun, 2024).

To conclude, the presented data allows viewing the phenomenon of voting for the first time through the prism of being a psychological threshold experience rather than the start of the process of forming habits (Plutzer, 2002). The creation of identity, emotional evaluation, consistency seeking, and judgment of efficacy form dimensions of a single experience. Furthermore, by using concepts from political psychology to interpret the themes developed through reflexive thematic analysis, this study shows how qualitative research can provide new insights into processes that have often been examined through surveys and experiments.

In order to expand research within this field in the future, the possible link between the identity associated with belonging to a specific generation and partisan perspective should be explored. In this case, the issue of emotional ambivalence in electoral processes and its connection to perceptions of political efficacy will be explored more thoroughly and the findings will be enriched. In addition, comparative research conducted in polarized political environment and contexts characterized by economic uncertainty will be helpful in assessing whether the findings are generational phenomena or peculiar only to the Turkish one.

## 7 Conclusion

Conceptualizing first-time voting behavior as a psychological threshold experience represents an important step in developing the theoretical basis of electoral participation. The aim of this research was to dispute the perception of first-time voting behavior either as something that simply becomes the start of a usual routine or as some kind of rational strategy. That is because first-time voting transcends both frameworks and appears to be an activity related to the formation of political identity, the evaluation of emotions and perceptions of political competence. In this particular case, voting among youth in the country under examination is closely intertwined with the political and economic situation. What is more, the construction of generational identity is directly linked with the feeling of economic uncertainty and distrust in institutions. Emotions that are triggered by such circumstances are not separate triggers that prompt voters to go to the polls. They are rather the result of the complicated combination of hopes and uncertainties, anxieties and sense of civic belonging.

This perspective reveals that the coexistence of a sense of civic duty and concerns about the responsiveness of the political system highlights how participation in electoral processes is shaped by current conditions. In this context, while participants may view voting behavior as valuable within democratic systems, their perceptions of the political impact of their votes remain closely linked to the responsiveness of the political system. By using reflexive thematic analysis within a political psychology framework, the present study complements research that has primarily examined voting behavior through survey research and quantitative measures by focusing on the meanings participants attach to their first voting experience. The fact that this study aimed to better understand how people position themselves politically the very first time they vote allows us to further examine how the first election experience may relate to future political involvement. Future research could follow the same people over many election cycles to learn more about how the perceptions that emerged in this study change over time. Thus, comparative research could be carried out in other democracies with political polarization or different kinds of political instabilities to find out whether the results of this study are unique for Türkiye or rather generational.

## Data Availability

The datasets presented in this article are not readily available because the qualitative interview data are subject to ethical, confidentiality, and participant privacy restrictions. De-identified data supporting the findings and relevant coding materials may be made available upon reasonable request, where appropriate and subject to these restrictions. Requests to access the datasets should be directed to Fatih Yaman, fatih.yaman@cbu.edu.tr.

## References

[ref1] Balcı Ş. İldız S. Okur M. S. (2026). Sosyal medya kullanımının politik okuryazarlık üzerindeki etkisi: 31 Mart 2024 yerel seçimlerinde genç seçmenler araştırması. Hitit Sosyal Bilimler Dergisi 19, 301–326. doi: 10.17218/hititsbd.1711591

[ref2] Beasley R. K. Joslyn M. R. (2001). Cognitive dissonance and post-decision attitude change in six presidential elections. Polit. Psychol. 22, 521–540. doi: 10.1111/0162-895X.00252

[ref3] Bhatti Y. Hansen K. M. (2012). Leaving the nest and the social act of voting: turnout among first-time voters. J. Elect. Public Opin. Parties 22, 380–406. doi: 10.1080/17457289.2012.721375

[ref4] Blais A. (2006). What affects voter turnout? Annu. Rev. Polit. Sci. 9, 111–125. doi: 10.1146/annurev.polisci.9.070204.105121

[ref5] Brader T. (2005). Striking a responsive chord: how political ads motivate and persuade voters by appealing to emotions. Am. J. Polit. Sci. 49, 388–405. doi: 10.1111/j.0092-5853.2005.00130.x

[ref6] Braun V. Clarke V. (2006). Using thematic analysis in psychology. Qual. Res. Psychol. 3, 77–101. doi: 10.1191/1478088706qp063oa

[ref7] Braun V. Clarke V. (2019). Reflecting on reflexive thematic analysis. Qual. Res. Sport, Exerc. Health 11, 589–597. doi: 10.1080/2159676X.2019.1628806

[ref8] Eckstein K. Miklikowska M. Šerek J. Noack P. Koerner A. (2024). Activating effects of elections: changes in young voters’ political engagement over the course of an election year. Front. Polit. Sci. 6:1302686. doi: 10.3389/fpos.2024.1302686

[ref9] Fox S. (2024). Social action as a route to the ballot box: can youth volunteering reduce inequalities in turnout? Eur. J. Polit. Res. 63, 111–131. doi: 10.1111/1475-6765.12586

[ref10] Greene S. (2004). Social identity theory and party identification. Soc. Sci. Q. 85, 136–153. doi: 10.1111/J.0038-4941.2004.08501010.X

[ref11] Henn M. Foard N. (2014). Social differentiation in young people’s political participation: the impact of social and educational factors on youth political engagement in Britain. J. Youth Stud. 17, 360–380. doi: 10.1080/13676261.2013.830704

[ref12] Huddy L. (2001). From social to political identity: a critical examination of social identity theory. Polit. Psychol. 22, 127–156. doi: 10.1111/0162-895X.00230

[ref13] Karp J. A. Banducci S. A. (2008). Political efficacy and participation in twenty-seven democracies: how electoral systems shape political behaviour. Br. J. Polit. Sci. 38, 311–334. doi: 10.1017/S0007123408000161

[ref14] Kocaman R. Coşgun M. (2024). A qualitative research to explore how young voters perceive politicians’ use of social media. Alanya Akademik Bakış 8, 751–765. doi: 10.29023/alanyaakademik.1445440

[ref15] Lau R. R. Redlawsk D. P. (2001). Advantages and disadvantages of cognitive heuristics in political decision making. Am. J. Polit. Sci. 45, 951–971. doi: 10.2307/2669334

[ref16] Marcus G. E. MacKuen M. Neuman W. R. (2011). Parsimony and complexity: developing and testing theories of affective intelligence. Polit. Psychol. 32, 323–336. doi: 10.1111/j.1467-9221.2010.00806.x

[ref17] Mason L. (2015). “I disrespectfully agree”: the differential effects of partisan sorting on social and issue polarization. Am. J. Polit. Sci. 59, 128–145. doi: 10.1111/ajps.12089

[ref18] Niemi R. G. Craig S. C. Mattei F. (1991). Measuring internal political efficacy in the 1988 National Election Study. Am. Polit. Sci. Rev. 85, 1407–1413. doi: 10.2307/1963953

[ref19] Oral U. (2023). The use of media by Turkey’s generation Z as a source for political information. OPUS Toplum Araştırmaları Dergisi 20, 162–173. doi: 10.26466/opusjsr.1214406

[ref20] Öz Döm Ö. Bingöl Y. (2021). Political participation of youth in Turkey: social media as a motivation. İnsan ve Toplum Bilimleri Araştırmaları Dergisi 10, 605–625. doi: 10.15869/itobiad.804311

[ref21] Plutzer E. (2002). Becoming a habitual voter: inertia, resources, and growth in young adulthood. Am. Polit. Sci. Rev. 96, 41–56. doi: 10.1017/S0003055402004227

[ref22] Riker W. H. Ordeshook P. C. (1968). A theory of the calculus of voting. Am. Polit. Sci. Rev. 62, 25–42. doi: 10.2307/1953324

[ref23] Sarıhan Z. Taşcıoğlu R. (2024). Türkiye’de Gençlerin Dijital Siyasal Katılımlarına Yönelik Bir Araştırma. Etkileşim 7, 46–80. doi: 10.32739/etkilesim.2024.7.14.260

[ref24] Smets K. Van Ham C. (2013). The embarrassment of riches? A meta-analysis of individual-level research on voter turnout. Elect. Stud. 32, 344–359. doi: 10.1016/j.electstud.2012.12.006

[ref25] Taber C. S. Lodge M. (2006). Motivated skepticism in the evaluation of political beliefs. Am. J. Polit. Sci. 50, 755–769. doi: 10.1111/j.1540-5907.2006.00214.x

[ref26] Tajfel H. (1974). Social identity and intergroup behaviour. Soc. Sci. Inf. 13, 65–93. doi: 10.1177/053901847401300204

[ref27] Tong A. Sainsbury P. Craig J. (2007). Consolidated criteria for reporting qualitative research (COREQ): a 32-item checklist for interviews and focus groups. Int. J. Qual. Health Care 19, 349–357. doi: 10.1093/intqhc/mzm042, 17872937

[ref28] Toros S. Toros E. (2022). Social media use and political participation: the Turkish case. Turk. Stud. 23, 450–473. doi: 10.1080/14683849.2021.2023319

[ref29] Valentino N. A. Brader T. Groenendyk E. W. Gregorowicz K. Hutchings V. L. (2011). Election night’s alright for fighting: the role of emotions in political participation. J. Polit. 73, 156–170. doi: 10.1017/S0022381610000939

