# Supplementary material for: First-time voters in Türkiye: a thematic analysis from a political psychology perspective
Source: Front Psychol. 2026 Oct 28;17:1976074. doi: 10.3389/fpsyg.2026.1976074 (PMC13752182; doi:10.3389/fpsyg.2026.1976074)
Supplement: Supplementary file 1 [file Supplementary_file_1.DOCX]

**SUPPLEMENTARY FILE 1**

**COREQ (Consolidated Criteria for Reporting Qualitative Research) 32-item Checklist** (Tong, Sainsbury & Craig, 2007)

| **No** | **Item** | **Description** | **Reported in Manuscript** |
| --- | --- | --- | --- |
| **Domain 1: Research Team and Reflexivity** | | | |
| 1 | Interviewer/facilitator | Interviews were conducted jointly by the first author (male academic) and the third author (female postgraduate student). | Method 4.3 |
| 2 | Credentials | First and second authors: academics in political science; third author: postgraduate student in political science. | Method 4.3 |
| 3 | Occupation | First and second authors: academics in political science; third author: postgraduate student in political science. | Method 4.3 |
| 4 | Gender | First and second authors: male; third author: female. | Method 4.3 |
| 5 | Experience and training | All the researchers have academic background in political science and experience in qualitative research. | Method 4.3 |
| 6 | Relationship established | Some participants from Manisa Celal Bayar University were acquainted with researchers academically; others had no prior relationship. | Method 4.3 |
| 7 | Participant knowledge of interviewer | Participants were informed about researchers’ roles and research purpose prior to interviews. | Method 4.3 & 4.6 |
| 8 | Interviewer characteristics | Researchers acknowledged their academic background and scholarly interest in youth political participation; reflexive awareness was maintained throughout data collection and analysis. | Method 4.3 & 4.5 |
| **Domain 2: Study Design** | | | |
| 9 | Methodological orientation | Qualitative design using reflexive thematic analysis, grounded in a constructivist orientation. | Method 4.2 |
| 10 | Sampling | Maximum variation purposive sampling. | Method 4.3 |
| 11 | Method of approach | Face-to-face in-depth interviews. | Method 4.3 |
| 12 | Sample size | 26 participants. | Method 4.3 |
| 13 | Non-participation | No reported refusals or dropouts. | Method 4.3 |
| 14 | Setting of data collection | Interviews were conducted face-to-face in the first author’s office at the Faculty of Economics and Administrative Sciences, Manisa Celal Bayar University, which provided a quiet and private setting. | Method 4.3 |
| 15 | Presence of non-participants | No non-participants present during interviews. | Method 4.3 |
| 16 | Description of sample | Detailed demographic table (university, department, gender, birth year, elections voted). | Table 1 |
| 17 | Interview guide | Semi-structured interview guide developed and pilot-tested prior to data collection; the complete guide is provided in Supplementary File 2. | Method 4.3 |
| 18 | Repeat interviews | No repeat interviews conducted. | Method 4.3 |
| 19 | Audio/visual recording | Interviews audio-recorded and transcribed verbatim. | Method 4.3 & 4.4 |
| 20 | Field notes | Field notes and analytic memos were taken throughout data collection and analysis. | Method 4.4 |
| 21 | Duration | Interviews lasted approximately 30-40 minutes. | Method 4.3 |
| 22 | Data saturation | Data collection concluded after 26 interviews, when later interviews were producing increasingly recurrent accounts. This was treated as a pragmatic indication of dataset adequacy rather than as evidence that all possible themes had been exhausted. | Method 4.5 |
| 23 | Transcripts returned | Full transcripts were not returned. Selected participants were contacted during the analytical process to provide reflections on preliminary thematic interpretations. | Method 4.5 |
| **Domain 3: Analysis and Findings** | | | |
| 24 | Number of data coders | The first and second authors engaged in iterative analytical discussions during theme development and refinement. These discussions considered alternative interpretations and theme boundaries rather than establishing inter-coder agreement. | Method 4.5 |
| 25 | Description of coding tree | Themes, categories, codes, and illustrative participant extracts are presented in Table 2. | Method 4.4 & Table 2 |
| 26 | Derivation of themes | Initial codes were developed inductively and candidate themes were developed and refined iteratively through repeated movement between coded segments, individual transcripts, and the dataset as a whole. | Method 4.4 |
| 27 | Software | MAXQDA 26 used for analysis. | Method 4.4 |
| 28 | Participant checking | Selected participants provided reflections on preliminary thematic interpretations. Their responses were treated as an additional interpretive perspective rather than as verification of a single correct interpretation. | Method 4.5 |
| 29 | Quotations presented | Participant quotations from a broad range of participants are included in the Findings section with identifiers (P1–P26). | Findings |
| 30 | Data and findings consistent | Themes are supported by verbatim participant extracts and linked to the reported patterns of meaning. | Findings |
| 31 | Clarity of major themes | Major themes clearly presented in structured Findings section. | Findings |
| 32 | Clarity of minor themes | Variations and less frequent codes are reported with descriptive frequency indicators. Frequencies are not treated as indicators of thematic importance. | Method 4.4 & Findings |
