# Supplementary material for: First-time voters in Türkiye: a thematic analysis from a political psychology perspective
Source: Front Psychol. 2026 Oct 28;17:1976074. doi: 10.3389/fpsyg.2026.1976074 (PMC13752182; doi:10.3389/fpsyg.2026.1976074)
Supplement: Supplementary file 2 [file Supplementary_file_2.DOCX]

**Supplementary File 2**

**Semi-Structured Interview Guide**

*First-time voters in Türkiye: a thematic analysis from a political psychology perspective*

**Basic Interview Information**

Interview date:

Interview duration:

Participant’s department:

Participant’s university:

Participant’s age:

Participant’s gender:

**Core Interview Questions**

1. What does the term Generation Z mean to you? How would you describe Generation Z?

2. What was voting like as an experience? Why did you vote?

3. What were the main factors that influenced your voting behaviour? What roles did your political views, candidates’ promises, election campaigns, media, social environment, and social media play?

4. What positive or negative situations did you observe during the election process? How did these situations affect your political participation or preferences?

5. How do you evaluate Generation Z’s expectations from politics and politicians?

6. What sources or methods do you use to increase your political awareness? What role do these sources or methods play in shaping your political views?

7. How do you evaluate the different political attitudes or behaviours of Generation Z that you observe in your social environment or on social media?

8. What suggestions would you make to political parties and candidates regarding policies or discourse directed toward Generation Z? What can politicians do to respond better to young people’s expectations?

**Flexible Follow-up Probes**

Because the interviews were semi-structured, follow-up questions were used flexibly when needed to clarify participants’ responses, invite further explanation or examples, and explore issues raised during the interview in greater depth. The wording of these probes was not standardized and varied according to participants’ responses.

**Closing Questions**

1. Is there any issue that we have not discussed but that you would like to add?

2. If clarification is needed at a later stage, may we contact you again?
